# circAMOTL1L Suppresses Renal Cell Carcinoma Growth by Modulating the miR-92a-2-5p/KLLN Pathway

**DOI:** 10.1155/2021/9970272

**Published:** 2021-10-04

**Authors:** Ling Gao, Xian Shao, Qingqing Yue, Weifei Wu, Xuejuan Yang, Xiaolei He, Limin Li, Fujun Hou, Ruonan Zhang

**Affiliations:** ^1^Department of Oncology, Kaifeng Central Hospital, Kaifeng, Henan, China; ^2^Department of Anesthesiology, The No. 4 Hospital of Shijiazhuang, Shijiazhuang, Hebei, China; ^3^School of Chinese Integrative Medicine, Hebei Medical University, Shijiazhuang, Hebei, China; ^4^Clinical Laboratory, Handan First Hospital, Handan, Hebei, China; ^5^Department of Foreign Nursing, Chengde Nursing Vocational College, Chengde, Hebei, China

## Abstract

Accumulating evidence indicates that the dysregulation of circular RNAs (circRNAs) contributes to tumor progression; however, the regulatory functions of circRNAs in renal cell carcinoma (RCC) remain largely unknown. In this study, the function and underlying mechanism of circAMOTL1L in RCC progression were explored. qRT-PCR showed the downregulation of circAMOTL1L in RCC tissues and cell lines. The decrease in circAMOTL1L expression correlated with the tumor stage, metastasis, and poor prognosis in patients with RCC. Functional experiments revealed that circAMOTL1L inhibited cell proliferation and increased apoptosis in RCC cells. Subcutaneous implantation with circAMOTL1L-overexpressing cells in nude mice decreased the growth ability of the xenograft tumors. Mechanistically, circAMOTL1L served as a sponge for miR-92a-2-5p in upregulating KLLN (killin, p53-regulated DNA replication inhibitor) expression validated by bioinformatics analysis, oligo pull-down, and luciferase assays. Further, reinforcing the circAMOTL1L–miR-92a-2-5p–KLLN axis greatly reduced the growth of RCC *in vivo*. Conclusively, our findings demonstrate that circAMOTL1L has an antioncogenic role in RCC growth by modulating the miR-92a-2-5p–KLLN pathway. Thus, targeting the novel circAMOTL1L–miR-92a-2-5p–KLLN regulatory axis might provide a therapeutic strategy for RCC.

## 1. Introduction

Renal cell carcinoma (RCC) is a common malignant tumor of the human urinary system and the second leading cause of death among patients with urologic tumors, accounting for approximately 3% of all adult malignancies [1–3]. Although partial and radical nephrectomy is the most effective treatment for early-stage or localized RCC, approximately 30% of patients with RCC experience recurrence and metastasis [4, 5]. Owing to the insensitivity to radiotherapy and chemotherapy, the prognosis of patients with RCC is poor and the 5-year survival rate of advanced RCC is <10% [6, 7]. However, the pathogenesis of RCC remains to be clarified. Thus, novel molecular mechanisms underlying RCC progression need to be further explained to explore more effective therapeutic strategies for RCC.

Circular RNAs (circRNAs) are a novel class of single-stranded noncoding RNA characterized by covalently closed loops mostly generated from back-splicing of exons [8]. Compared with linear RNAs, circRNAs are structurally more stable and not easily degraded by RNases owing to their circular structure [9] and are widely identified in various eukaryotic cells [10, 11]. Accumulating studies have confirmed that circRNAs perform different critical regulatory activities in gene expression, including sponging miRNA [12], modulating alternative splicing [8], interacting with RNA binding proteins [13], and regulating gene transcription and protein-encoding ability [14]. Thus, circRNAs have been implicated in the pathogenesis of several human diseases, such as cancer, Alzheimer's disease, and cardiac diseases [15–18]. Particularly, accumulating evidence suggests that circRNAs play critical roles in tumor progression by regulating the proliferation, apoptosis, invasion, and metastasis of tumor cells [19–21] and are gradually being expected to become valuable prognostic biomarkers and therapeutic targets for malignant tumors. Recently, circRNAs have been increasingly reported to participate in RCC tumorigenesis. circSDHCs promote the proliferation and metastasis of RCC via the CDKN3/E2F1 axis [22]. circPVT1 promotes progression of ccRCC by sponging miR-145-5p and regulating TBX15 expression [23]. circPRRC2A promotes TRPM3-induced epithelial-mesenchymal transition of RCC through sponging miR-514a-5p and miR-6776-5p [24]. However, the exact function and underlying mechanism of circRNAs in RCC remain largely unclear.

circAMOTL1L (circBase ID: hsa_circ_0000350) is derived from back-splicing of the Amotl1 mRNA (from exon-2 to exon-3) and is located on chr11:94,528,176-94,533,477; it has been reported to exhibit a tumor suppressor characteristic in prostate cancer [25]. Here, we identified that circAMOTL1L is downregulated in RCC tissues and cell lines and is negatively associated with the growth of RCC cells. Mechanistically, circAMOTL1L served as a sponge of miR-92a-2-5p, thereby upregulating KLLN expression and inducing RCC growth inhibition. Conclusively, circAMOTL1L may serve as an antioncogene in RCC progression, and a novel regulatory network involving the circAMOTL1L–miR-92a-2-5p–KLLN axis might serve as a therapeutic strategy for RCC.

## 2. Materials and Methods

### 2.1. Clinical Sample Collection

All 51 pairs of RCC tissues and matched adjacent noncancerous tissues were collected from patients with RCC who had undergone surgical resection at the Kaifeng Central Hospital from 2015 to 2018. According to the histological classification, all RCC tissues were of clear cell RCC (ccRCC). All patients with RCC were pathologically diagnosed and received no preoperative treatment. This study protocol was permitted by the Ethics Committee of the Kaifeng Central Hospital, and all patients provided their informed consent.

### 2.2. Cell Culture and Transfection

The human renal tubular epithelial cell line (HK-2) and human RCC cell lines (786-O, ACHN, Caki1, Caki2, and 769-P) were obtained from the American Type Culture Collection (Manassas, VA, USA). Cells were cultured in RPMI 1640 medium containing 10% FBS (Gibco, Beijing, China), penicillin (100 U/ml), and streptomycin (100 *μ*g/ml) at 37°C with 5% CO_2_. Cell transfection was performed with Lipofectamine 2000 (Invitrogen) based on the manufacturer's instructions. The miR-92a-2-5p mimics, anti-miR-92a-2-5p, and their respective control RNAs were purchased from GenePharma (Shanghai, China). si-KLLN was purchased from Santa Cruz Biotechnology. circAMOTL1L overexpression and luciferase reporter vectors were synthesized by Biocaring Biotechnology Co., Ltd. (Shijiazhuang, China).

### 2.3. Plasmid and Lentivirus Expression Vector Constructs

The full-length sequence of circAMOTL1L was inserted into the pcDNA3.1 vector (Vazyme Biotech Co., Ltd.). WT or mutant circAMOTL1L sequences or 3′ UTR sequences of KLLN containing the WT or mutated miR-92a-2-5p binding site were inserted into the pmir-GLO Dual-Luciferase miRNA Target Expression Vector (Promega Corp., Madison, WI, USA).  Table 1 shows oligos for plasmid construction, probe, siRNA, and biotinylated oligos. Lentivirus encoding circAMOTL1L (LV-circAMOTL1L), anti-miR-92a-2-5p (LV-anti- miR-92a-2-5p), and LV control (LV-Ctl) was entrusted to Hanbio Co., Ltd (Shanghai, China).

### 2.4. qRT-PCR Analysis

Total RNA was extracted from clinical samples by treating cultured cells with the TRIzol reagent (Invitrogen). NanoDrop 2000 was used to determine RNA quality. To determine circAMOTL1L expression, the M-MLV First-Strand Kit (Life Technologies) and Platinum SYBR Green qPCR Super Mix UDG Kit (Invitrogen) were used on the ABI 7500 FAST system (Life Technologies). The miScripIIRT kit (Qiagen) and miScript SYBR Green PCR kit (Qiagen) were used to quantify miRNA expression. GAPDH was used as an internal control of circAMOTL1L, and U6 was used as an internal control of microRNAs. Relative transcript expression levels were calculated using the 2‐ΔΔCt method. Supplementary Table 1 lists the sequences of primers.

### 2.5. Cell Proliferation Assay

Cell proliferation was detected by Cell Counting Kit-8 (CCK-8) (Dojindo, Japan). Transfected 786-O cells were cultured in a 96-well plate (2 × 10^3^ per well) for 24, 48, 72, and 96 h. Next, 10 ml CCK-8 solution was added to each well and incubated for 2 h. The optical density value of each well was measured using a microplate reader (Bio-Rad, USA).

### 2.6. Apoptosis Assay

An apoptosis assay was performed using the TUNEL Apoptosis Detection kit (Yisheng, Shanghai, China) according to the specified instructions. DAPI (5 mg/ml) was used to stain the cell nuclei. Samples were detected using a Leica DM6000B microscope and digitized using LAS V.4.4 software. The TUNEL-positive rate reflecting the degree of apoptosis was calculated by enumerating the total number of TUNEL-stained nucleus divided by total DAPI-positive nucleus.

### 2.7. Biotin-Oligo Pull-Down Analysis

To find the interactions between circAMOTL1L and miRNAs, biotin-oligo pull-down analysis was performed as previously described.  Table 1 lists the biotin-labeled oligonucleotide probes against circAMOTL1L or miRNAs (GenePharma Co., Shanghai).

### 2.8. Luciferase Assay

For circAMOTL1L-miRNA luciferase assays, 786-O cells were cotransfected with the miR-92a-2-5p mimic (GenePharma; Shanghai) or Ctl mimic (200 pmol) combined with 100 ng circAMOTL1L-luciferase reporter (wt or mut) or an empty vector. For miR-92a-2-5p-KLLN luciferase assays, 786-O cells were cotransfected with the miR-92a-2-5p mimic or Ctl mimic combined with the KLLN-3′ UTR-luciferase reporter (wt or mut). Luciferase activity was examined using a Dual-Glo Luciferase Assay System (Promega, Madison, WI) with a flash and glow (LB955, Berthold Technologies). The specific activity was described as firefly luciferase activity normalized to Renilla luciferase activity.

### 2.9. Western Blotting

RCC cells were lysed, and xenograft tissues were homogenized using a RIPA lysis buffer (Beyotime, Shanghai, China) with phenylmethanesulfonyl fluoride (PMSF). Total protein was separated by 10% SDS-PAGE and electrotransferred to a PVDF membrane (Millipore, Billerica, MA). Membranes were blocked with 5% milk in the TTBS buffer for 2 h at room temperature and incubated with a primary antibody (anti-KLLN Ab-16934, anti-*β*-actin sc-47778) overnight at 4°C. Membranes were then incubated with a horseradish peroxidase-conjugated secondary antibody for 2 h at room temperature. The protein blots were treated with Immobilon™ Western (Millipore) and then evaluated using the ECL detection system (Vilber Lourmat).

### 2.10. Xenograft Model

The *in vivo* study was performed in accordance with the Code of Ethics of the World Medical Association and approved by the Institutional Animal Care and Use Committee of Hebei Medical University. The xenograft model was established as described previously (1). Briefly, 4–6-week-old male BALB/c nude mice (Vital River Laboratory Animal Technology Co., Ltd. Beijing) were subcutaneously injected in the back on the right side with 5 × 10^7^ 786-O cells stably expressing circAMOTL1L, miR-92a-2-5p, or both; 6 weeks after injection, mice were sacrificed to measure tumor weight. Proteins from xenograft tissues were extracted to detect gene expression.

### 2.11. Statistical Analyses

The applicable statistical methods were used depending on the type of data. Student's *t*-test was performed for comparisons between two groups. Linear correlations were evaluated using the Pearson correlation analysis. A chi-squared test was used to analyze the correlation among the clinicopathological characteristics of patients with RCC and circAMOTL1L expression. The Kaplan–Meier method was used to assess the overall survival and disease-free survival curves. Pearson correlation analysis was used to analyze the correlations between the groups. All data from independent repeated trials are presented as means ± SEM. A *p* value of <0.05 was considered statistically significant. Statistical analysis was performed using GraphPad Prism 7.0 software.

## 3. Results

### 3.1. circAMOTL1L Expression Is Downregulated in Human RCC Tissues and Cell Lines and Correlates with Poor Prognosis

**Figure 1:**
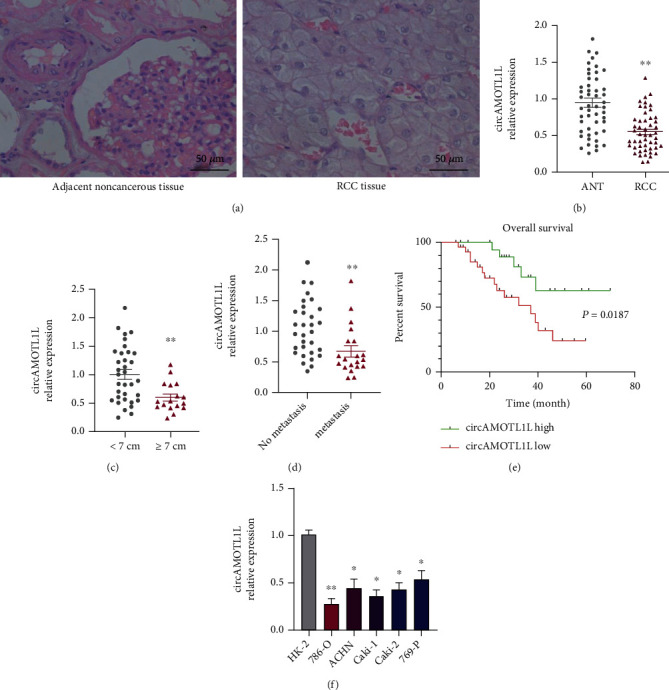
circAMOTL1L is downregulated in RCC tissues and cells and correlates with poor prognosis. (a) Hematoxylin and eosin staining of RCC tissues and adjacent noncancerous tissues. (b) qRT-PCR examined circAMOTL1L expression in 51 paired RCC tissues and adjacent noncancerous tissues. Normalized to GAPDH. ^∗∗^*p* < 0.01 vs. normal adjacent noncancerous tissues. (c) The circAMOTL1L expression levels in RCC tissues with different tumor sizes. ^∗∗^*p* < 0.01 vs. tumor sizes < 7 cm. (d) circAMOTL1L expression in patients with RCC with or without metastasis. ^∗∗^*p* < 0.01 vs. no metastasis. (e) Kaplan–Meier analysis of the overall survival of patients with RCC having high and low expression of circAMOTL1L. ^∗^*p* < 0.05. (f) qRT-PCR examined circAMOTL1L expression in the human renal tubular epithelial cell line HK-2 and five RCC cell lines. ^∗^*p* < 0.05, ^∗∗^*p* < 0.01 vs. HK2 cells. Graph bars represent mean ± SEM of three independent experiments.

Fifty-one pairs of RCC tissues and adjacent noncancerous tissues were collected to detect circAMOTL1L expression using qRT-PCR. The circAMOTL1L expression level significantly decreased in RCC tissues compared with adjacent noncancerous tissues (Figures 1(a) and 1(b)). Moreover, the circAMOTL1L expression level in RCC samples negatively correlated with tumor size, tumor stage, and metastasis, but not with age and sex (Figures 1(c) and 1(d), Table 1). In addition, low circAMOTL1L expression in patients with RCC was associated with a lower overall survival rate than high circAMOTL1L expression (Figure 1(e)). Next, we evaluated circAMOTL1L expression in different RCC cell lines. Results of qRT-PCR showed that the circAMOTL1L expression level was lower in RCC cell lines, particularly in 786-O and Caki-1 cell lines, than in normal kidney cells (HK-2) (Figure 1(f)). These findings indicate that circAMOTL1L might act as an antioncogene in RCC.

### 3.2. circAMOTL1L Inhibits Cell Proliferation and Promotes Apoptosis *In Vitro* and Suppresses Tumor Growth *In Vivo*

**Figure 2:**
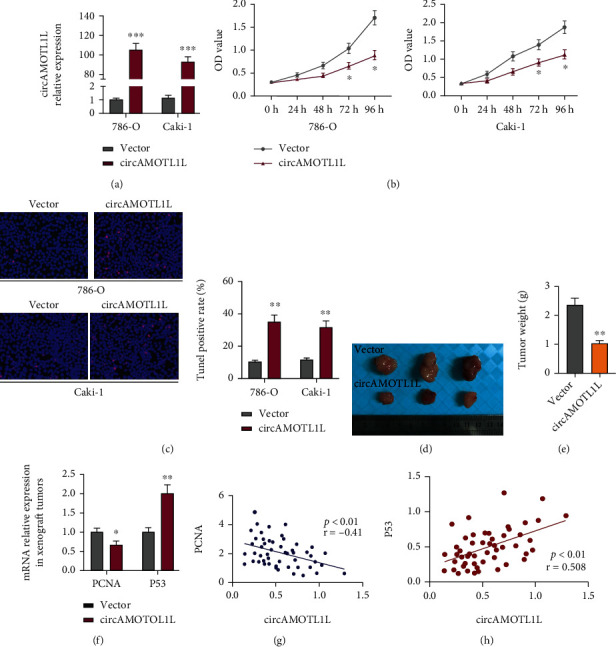
circAMOTL1L inhibits cell proliferation and promotes apoptosis *in vitro* and suppresses tumor growth *in vivo.* (a) qRT-PCR examined the circAMOTL1L expression level in 786-O and Caki-1 cells after transfecting with pcDNA-circAMOTL1L or vector control. ^∗∗∗^*p* < 0.001 vs. vector control. (b) CCK-8 assay examined the proliferation of 786-O and Caki-1 cells transfected with pcDNA-circAMOTL1L or vector control. ^∗^*p* < 0.05 vs. vector control. (c) TUNEL assay detected apoptosis of 786-O and Caki-1 cells after transfecting with pcDNA-circAMOTL1L or vector control. ^∗∗^*p* < 0.01 vs. vector control (scale bars = 50 *μ*m). The above graph bars represent mean ± SEM of three independent experiments. (d, e) Representative dissected xenograft tumors from nude mice subcutaneously injected with 786-O cells engineered to stably overexpress circAMOTL1L (LV-circAMOTL1L) for 3 weeks (LV-Ctl as the negative control) and corresponding weight measurement. ^∗∗^*p* < 0.01 vs. LV-Ctl. (f) qRT-PCR examined PCNA and P53 expression in xenograft tumors. ^∗^*p* < 0.05, ^∗∗^*p* < 0.01 vs. LV-Ctl (*n* = 6 in each group). (g, h) The Pearson correlation analyzed the relationship between PCNA mRNA and circAMOTL1L (*p* < 0.01, *r* = −0.41) and P53 mRNA and circAMOTL1L (*p* < 0.01, *r* = 0.508).

To investigate the function of circAMOTL1L in RCC cells, 786-O and Caki-1 cells were transfected with the circAMOTL1L overexpression plasmid (pcDNA-circAMOTL1L) or its empty vector. Observably, transfection with pcDNA-circAMOTL1L in 786-O and Caki-1 cells strikingly increased the circAMOTL1L expression level compared with the empty vector (Figure 2(a)). Then, CCK-8 and TUNEL assays were performed to examine cell proliferation and apoptosis. CCK-8 data revealed that circAMOTL1L overexpression suppressed the proliferation of 786-O and Caki-1 cells (Figure 2(b)). Meanwhile, circAMOTL1L overexpression significantly promoted the apoptosis of RCC cells (Figure 2(c)). Moreover, silencing circAMOTL1L expression facilitated the proliferation and inhibited the apoptosis of RCC cells (Supplementary Figures 1a–1c). However, the overexpression or depletion of circAMOTL1L had no effect on phenotypic transformation in HK-2 cells (Supplementary Figured 2a and 2b). To evaluate whether circAMOTL1L suppresses tumor growth *in vivo*, xenograft models were established by implanting 786-O cells engineered to stably overexpress circAMOTL1L (LV-circAMOTL1L) into nude mice. Six weeks after the xenograft, tumor development in the LV-circAMOTL1L group was significantly slower and the tumor weight was clearly lower than that in the control group (LV-Ctl) (Figures 2(d) and 2(e)). Next, we observed the proliferative and apoptotic phenotype *in vivo* by examining the expression of PCNA, P53, Bax, and bcl-2 in xenograft tumors. As shown in Figure 2(f), PCNA was downregulated and P53 was upregulated by implantation with circAMOTL1L-overexpressing 786-O cells in nude mice. Simultaneously, circAMOTL1L overexpression elevated Bax expression and reduced Bcl-2 expression (Supplementary Figure 3a). Furthermore, PCNA, P53, Bax, and bcl-2 expression levels were examined in RCC clinical samples. Correlation analysis revealed that the expression level of PCNA and bcl-2 was negatively correlated with that of circAMOTL1L, but the expression level of P53 and Bax was positively correlated with that of circAMOTL1L (Figures 2(g) and 2(h), Supplementary Figures 3b and 3c). These results suggested that circAMOTL1L prompted RCC cells to gain more apoptotic activity *in vivo.* Overall, circAMOTL1L may be responsible for RCC progression by regulating the proliferation and apoptosis of RCC cells.

### 3.3. circAMOTL1L Sponges miR-92a-2-5p in RCC Cells

**Figure 3:**
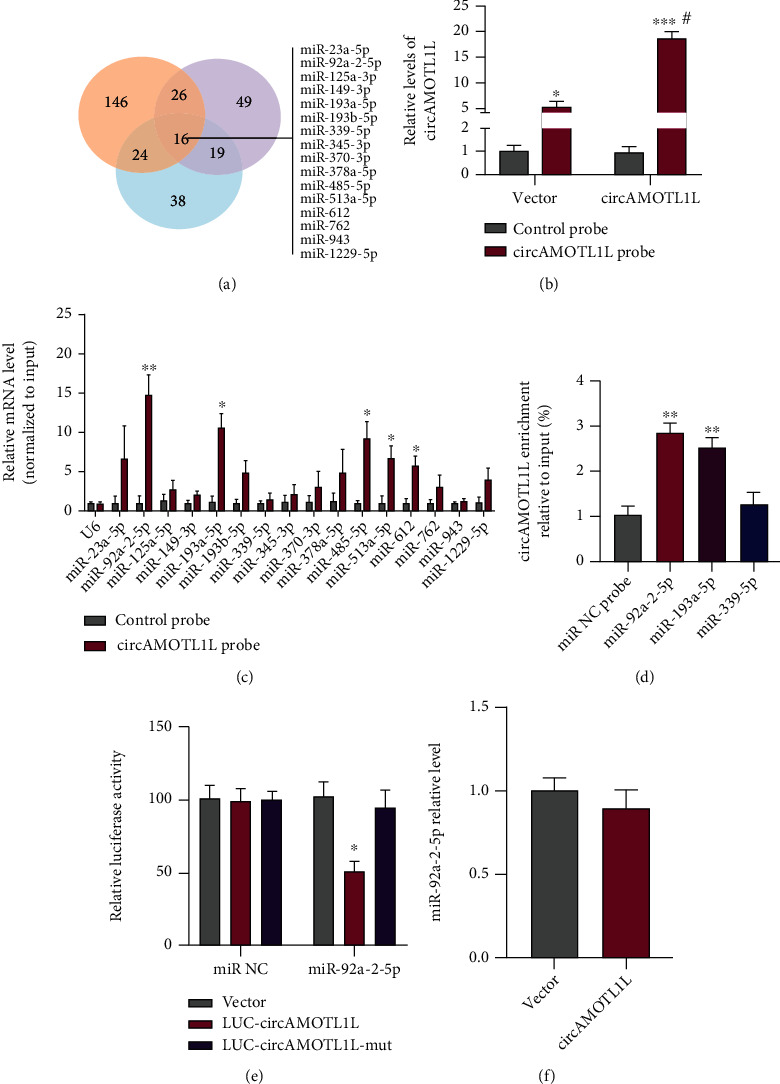
circAMOTL1L sponges miR-92a-2-5p in the RCC cell. (a) Venn diagram hybrid of target miRNA from three prediction programs (miRanda, RNAhybrid, and RNA22). (b) pcDNA-circAMOTL1L or empty vector was transfected into 786-O cells. qRT-PCR examined the pull-down efficiency of circAMOTL1L or NC probe. ^∗^*p* < 0.05, ^∗∗∗^*p* < 0.001, ^##^*p* < 0.01 vs. corresponding control. (c) qRT-PCR examined the relative expression of indicated miRNAs from pull-down precipitates. Normalized to control U6. ^∗^*p* < 0.05, ^∗∗^*p* < 0.01 vs. control probe. (d) qRT-PCR examined circAMOTL1L enrichment sedimented by biotin-labeled miR-92a-2-5p, miR-193a-5p, or miR-339-5p or NC probes. ^∗∗^*p* < 0.01 vs. NC probe. (e) Luciferase reporter assays examined luciferase activity of LUC-circAMOTL1L or LUC-circAMOTL1L-mut reporter constructs in 786-O cells cotransfected with the miR-92a-2-5p mimic or NC miR. ^∗^*p* < 0.05 vs. empty vector. (f) qRT-PCR examined miR-92a-2-5p expression in 786-O cells transfected with pcDNA-circAMOTL1L or empty vector. Graph bars represent mean ± SEM of three independent experiments.

Because circRNAs have a sponging effect on miRNA, we performed bioinformatics analysis to predict the potential binding of miRNAs to circAMOTL1L. The results of three target prediction programs (miRanda, RNAhybrid, and RNA22) revealed 16 putative cireAMOTL1L-binding miRNAs (Figure 3(a)). Next, the pull-down assay using a biotin-labeled circAMOTL1L probe was performed to examine miRNA expression in circAMOTL1L-overexpressed 786-O cells. qRT-PCR showed that the pull-down efficiency of circAMOTL1L significantly improved in circAMOTL1L-overexpressing cells compared with vector and control probe groups (Figure 3(b)). Next, the level of potential miRNAs in the precipitates that were pulled down with a circAMOTL1L biotin-labeled probe was detected by qRT-PCR. Apart from miR-193a-5p, a confirmed circAMOTL1L-binding miRNA in the previous study [25], miR-92a-2-5p, miR-485-5p, miR-513a-5p, and miR-612 were also clearly enriched in the precipitates (Figure 3(c)). Among these miRNAs, expression level of miR-92a-2-5p was more significantly enriched (*p* < 0.01) than that of other precipitated miRNAs. Moreover, circAMOTL1L harbors six supposed binding sites for miR-92a-2-5p (Supplementary Table 2). Therefore, we selected miR-92a-2-5p for further analyses. Subsequently, biotin-labeled miR-92a-2-5p, miR-193a-5p, or miR-339-5p were used to pull down circAMOTL1L, in which miR-193a-5p and miR-339-5p were used as positive and negative controls, respectively. The result from qRT-PCR showed that circAMOTL1L enrichment was notably elevated in the complexes sedimented by miR-92a-2-5p and miR-193a-5p but not miR-339-5p (Figure 3(d)). Furthermore, luciferase assays revealed that cotransfection with the circAMOTL1L-luciferase-reporter plasmid and miR-92a-2-5p mimic considerably reduced luciferase activity mediated by inserting a wild-type circAMOTL1L sequence and did not affect luciferase activity mediated by inserting miR-92a-2-5p binding site-mutated circAMOTL1L sequence (Figure 3(e)). These data indicate that circAMOTL1L could directly bind to miR-92a-2-5p. However, circAMOTL1L overexpression did not affect the miR-92a-2-5p expression level (Figure 3(f)). Collectively, these results reveal that circAMOTL1L serves as a sponge for miR-92a-2-5p without regulating miR-92a-2-5p expression.

### 3.4. miR-92a-2-5p Mediates the Regulation of circAMOTL1L in the Proliferation and Apoptosis in RCC Cells

**Figure 4:**
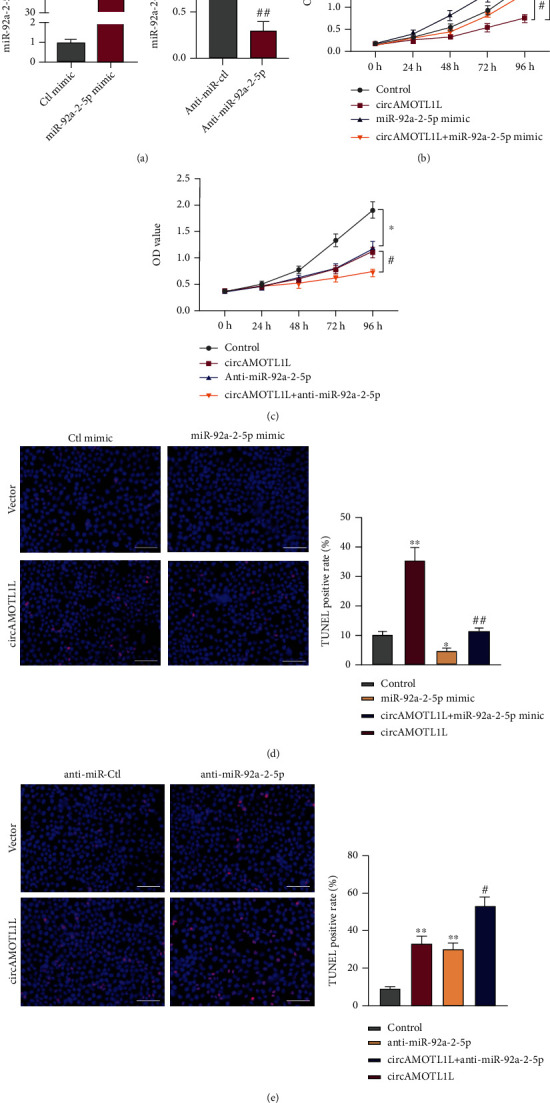
miR-92a-2-5p mediates the regulation of circAMOTL1L in the proliferation and apoptosis of RCC cells. (a) qRT-PCR examined miR-92a-2-5p expression in 786-O cells transfected with the miR-92a-2-5p mimic, anti-miR-92a-2-5p, or its corresponding control miR. ^∗∗∗^*p* < 0.001 vs. Ctl mimic; ^##^*p* < 0.01 vs. anti-miR-Ctl. (b) CCK-8 assay examined the proliferation of 786-O cells after transfection with pcDNA-circAMOTL1L or miR-92a-2-5p mimic or cotransfection with both. ^∗^*p* < 0.05 vs. control group; ^#^*p* < 0.05 vs. pcDNA-circAMOTL1L group. (c) CCK-8 assay examined cell proliferation in 786-O cells after transfection with pcDNA-circAMOTL1L or anti-miR-92a-2-5p or cotransfection with both. ^∗^*p* < 0.05 vs. control group; ^#^*p* < 0.05 vs. pcDNA-circAMOTL1L group. (d, e) TUNEL assay detected cell apoptosis in 786-O cells treated as (b) and (c), respectively. ^∗^*p* < 0.05, ^∗∗^*p* < 0.01 vs. control group; ^#^*p* < 0.05, ^##^*p* < 0.01 vs. pcDNA-circAMOTL1L group (scale bars = 50 *μ*m). Graph bars represent mean ± SEM of three independent experiments.

Furthermore, we tested the involvement of miR-92a-2-5p in cell proliferation and apoptosis regulated by circAMOTL1L. From Figure 4(a), transfection with the miR-92a-2-5p mimic strikingly increased, whereas transfection with anti-miR-92a-2-5p decreased the miR-92a-2-5p expression level in 786-O cells compared with their negative control. Next, 786-O cells were transfected with pcDNA-circAMOTL1L or miR-92a-2-5p mimic or cotransfected with both. CCK-8 and TUNEL data showed that the miR-92a-2-5p overexpression facilitated cell proliferation and suppressed apoptosis of 786-O cells. Moreover, the pcDNA-circAMOTL1L-induced inhibition of proliferation and promotion of apoptosis of cells were both abrogated by miR-92a-2-5p upregulation (Figures 4(b) and 4(d)). Next, we transfected 786-O cells with pcDNA-circAMOTL1L or anti-miR-92a-2-5p or both. Depletion of miR-92a-2-5p by its inhibitor decreased the cell proliferation and increased the apoptosis of 786-O cells (Figures 4(c) and 4(e)). However, the inhibition of proliferation and promotion of apoptosis of cells further improved after the knockdown of miR-92a-2-5p combined with circAMOTL1L overexpression. These data show that miR-92a-2-5p mediates the regulatory action of circAMOTL1L on the proliferation and apoptosis of RCC cells.

### 3.5. KLLN Is a Direct Target of miR-92a-2-5p in RCC Cells

**Figure 5:**
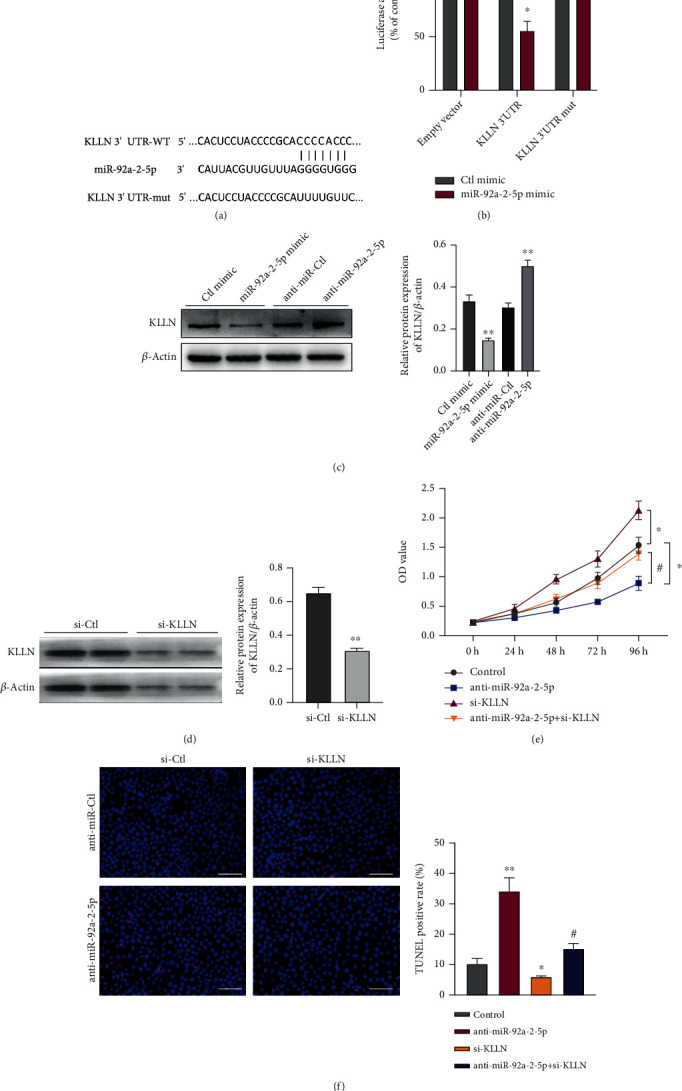
KLLN is a direct target of miR-92a-2-5p in the RCC cell. (a) Prediction of the miR-92a-2-5p binding site in KLLN 3′ UTR. (b) Luciferase reporter assays analyzed luciferase activity in 786-O cells after cotransfecting with the miR-92a-2-5p mimic or Ctl mimic and pmir-GLO vector containing the wild-type or mutated miR-92a-2-5p-binding site (mut) at KLLN 3′ UTR. ^∗^*p* < 0.05 vs. Ctl mimic. (c) Western blotting detected KLLN protein levels in 786-O cells transfected with the miR-92a-2-5p mimic, anti-miR-92a-2-5p, or its corresponding control miR. ^∗∗^*p* < 0.01 vs. corresponding control groups. (d) Western blotting detected KLLN protein levels in 786-O cells transfected with KLLN-specific siRNA (si-KLLN) or control siRNA (si-Ctl). ^∗∗^*p* < 0.01 vs. si-Ctl. (e) CCK-8 assay examined cell proliferation in 786-O cells after transfection with anti-miR-92a-2-5p or si-KLLN or cotransfection with both. ^∗^*p* < 0.05 vs. control group; ^#^*p* < 0.05 vs. anti-miR-92a-2-5p group. (f) TUNEL assay detected cell apoptosis in 786-O cells treated as (e). ^∗^*p* < 0.05, ^∗∗^*p* < 0.01 vs. control group; ^#^*p* < 0.05 vs. anti-miR-92a-2-5p group (scale bars = 50 *μ*m). Graph bars represent mean ± SEM of three independent experiments.

By predicting miR-92a-2-5p target gene using the TargetScan database (http://www.targetscan.org), we found that KLLN is known as a proapoptotic gene [26] contains complementary miR-92a-2-5p-binding sites in its 3′ UTR (Figure 5(a)). We further conducted the luciferase assay with the luciferase reporter plasmid expressing wild-type or mutant KLLN 3′ UTR. Data showed that the miR-92a-2-5p mimic apparently decreased the luciferase activity mediated by wild-type KLLN 3′ UTR, but not by the mutant one (Figure 5(b)). Further, we transfected 786-O cells with anti-miR-92a-2-5p, miR-92a-2-5p mimic, or their comparable control RNA. Western blotting showed that miR-92a-2-5p knockdown clearly increased KLLN protein expression, whereas miR-92a-2-5p overexpression significantly decreased KLLN protein expression (Figure 5(c)). These results suggest that miR-92a-2-5p inhibits the translation of KLLN by binding to 3′ UTR of KLLN. Subsequently, we used KLLN siRNAs to investigate the effect of KLLN on proliferation and apoptosis of cells and whether KLLN mediates the regulatory effect induced by miR-92a-2-5p. First, the transfection of si-KLLN in 786-O cells significantly reduced the KLLN protein expression compared with that in the control group (Figure 5(d)). Next, 786-O cells were transfected with anti-miR-92a-2-5p or si-KLLN or both. CCK-8 data display that KLLN depletion increased cell proliferation and further reversed the suppression of cell proliferation induced by miR-92a-2-5p knockdown (Figure 5(e)). Consistently, apoptosis rates were significantly decreased after KLLN depletion. However, the apoptosis facilitation after miR-92a-2-5p knockdown could be neutralized by cotransfection with si-KLLN (Figure 5(f)). These results indicate that miR-92a-2-5p exerts the promotion of cell proliferation and antiapoptotic effect by targeting KLLN.

### 3.6. circAMOTL1L–miR-92a-2-5p–KLLN Axis Is Involved in the Growth of RCC

**Figure 6:**
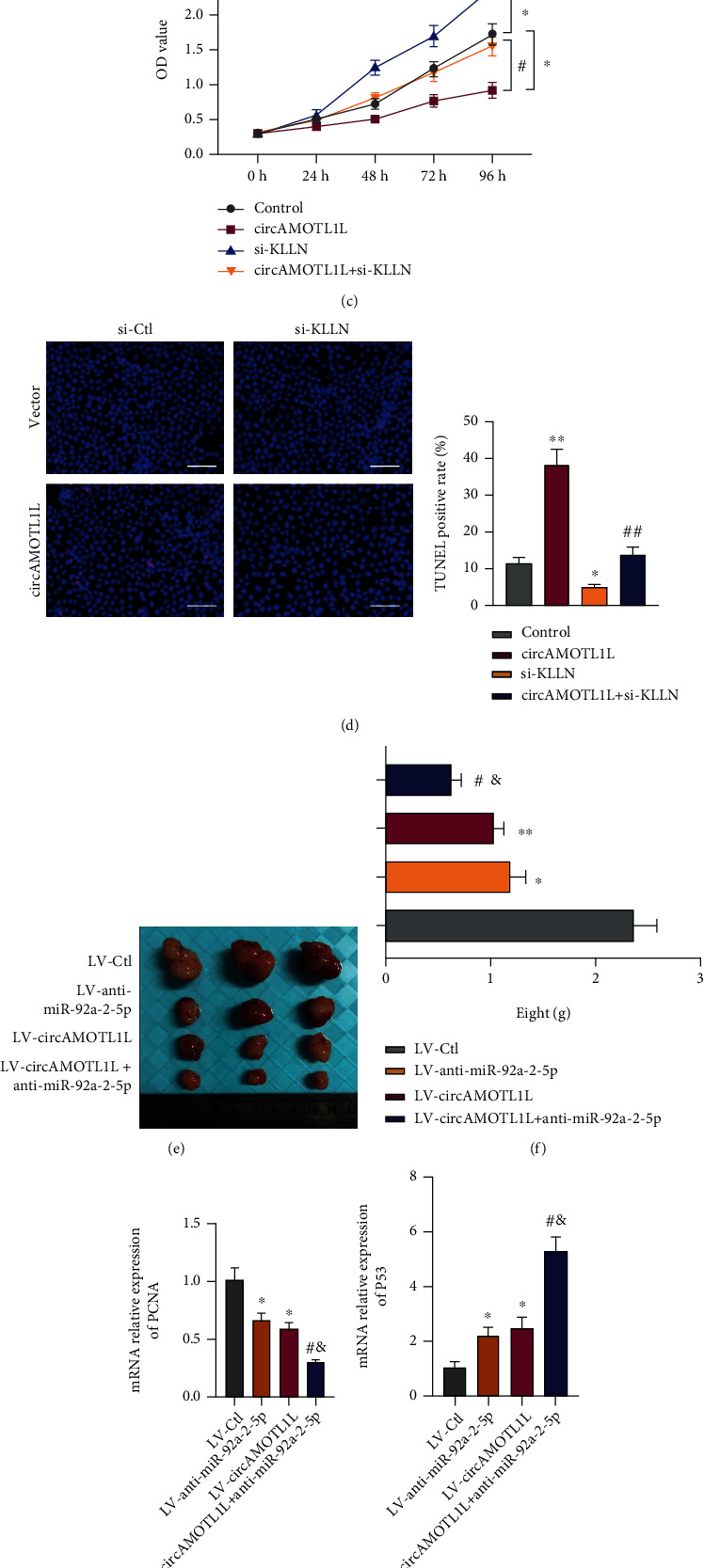
The circAMOTL1L–miR-92a-2-5p–KLLN axis is involved in the growth of RCC. (a, b) Western blotting detected KLLN protein levels in 786-O cells after transfection with pcDNA-circAMOTL1L, miR-92a-2-5p mimic, and anti-miR-92a-2-5p or cotransfection with pcDNA-circAMOTL1L and miR-92a-2-5p mimic or anti-miR-92a-2-5p. ^∗∗^*p* < 0.01 vs. control group; ^##^*p* < 0.01 vs. pcDNA-circAMOTL1L group. (c) CCK-8 assay examined cell proliferation in 786-O cells after transfection with pcDNA-circAMOTL1L or si-KLLN or cotransfection with both. ^∗^*p* < 0.05 vs. control group; ^#^*p* < 0.05 vs. pcDNA-circAMOTL1L group. (d) TUNEL assay detected apoptosis in 786-O cells treated as (c). ^∗^*p* < 0.05, ^∗∗^*p* < 0.01 vs. control group; ^##^*p* < 0.01 vs. pcDNA-circAMOTL1L group (scale bars = 50 *μ*m). The above graph bars represent mean ± SEM of three independent experiments. (e, f) Representative dissected xenograft tumors from nude mice subcutaneously injected with 786-O cells engineered to stably overexpress circAMOTL1L (LV-circAMOTL1L), anti-miR-92a-2-5p (LV-anti-miR-92a-2-5p), or both for 3 weeks (LV-Ctl as the negative control) and corresponding weight measurement. ^∗^*p* < 0.05, ^∗∗^*p* < 0.01 vs. LV-Ctl; ^#^*p* < 0.05 vs. LV-circAMOTL1L; ^&^*p* < 0.05 vs. LV-anti-miR-92a-2-5p. (g, h) qRT-PCR examined PCNA and P53 expression in xenograft tumors. ^∗^*p* < 0.05 vs. LV-Ctl; ^#^*p* < 0.05 vs. LV-circAMOTL1L; ^&^*p* < 0.05 vs. LV-anti-miR-92a-2-5p (*n* = 6 in each group). (i) Western blotting detected KLLN protein levels in xenograft tumors from the LV-circAMOTL1L plus LV-anti-miR-193a-5p infection group and LV-Ctl group. ^∗^*p* < 0.05 vs. LV-Ctl.

To elucidate the function of the circAMOTL1L–miR-92a-2-5p–KLLN axis on cell proliferation and apoptosis, rescue experiments were performed *in vitro*. First, we observed whether circAMOTL1L affects KLLN expression by interacting with miR-92a-2-5p. Western blotting revealed that circAMOTL1L overexpression upregulated KLLN protein level. However, the induced expression of KLLN by circAMOTL1L could be partly reversed by miR-92a-2-5p mimics and be further improved by the miR-92a-2-5p inhibitor (Figures 6(a) and 6(b)). Second, we assessed the relationship of circAMOTL1L and KLLN in the proliferation and apoptosis of RCC cells. KLLN depletion significantly reversed the antiproliferative and proapoptotic effects induced by circAMOTL1L overexpression (Figures 6(c) and 6(d)). The above data suggest that circAMOTL1L acts as a tumor suppressor by upregulating KLLN expression through sponging of miR-92a-2-5p in RCC cells. Moreover, we investigated the involvement of the circAMOTL1L–miR-92a-2-5p–KLLN axis in the RCC growth *in vivo*. 786-O cells stably transfected with LV-circAMOTL1L, LV-anti-miR-92a-2-5p, or both were injected into nude mice for establishing a xenograft model. Expectedly, the tumor growth was notably suppressed by implantation of circAMOTL1L-overexpressing 786-O cells or miR-92a-2-5p-depleted 786-O cells in nude mice compared with their corresponding control. circAMOTL1L overexpression combined with miR-92a-2-5p knockdown in 786-O cells further restrained the tumor growth in nude mice (Figure 6(e)). Correspondingly, the tumor weights of nude mice implanted with 786-O cells infected with LV-circAMOTL1L plus LV-anti-miR-92a-2-5p were further reduced compared with each of them alone (Figure 6(f)). Furthermore, circAMOTL1L overexpression or miR-92a-2-5p depletion decreased the expression of PCNA and Bcl-2 but increased the expression of P53 and Bax in the xenograft tumor, and this situation was further exacerbated by the superposition of the two (Figures 6(g) and 6(h), Supplementary Figures 4a and 4b). Next, we detected the KLLN protein levels in xenograft tissues by western blotting, and the results showed that KLLN protein levels significantly increased in xenograft tumors when circAMOTL1L overexpression was combined with miR-92a-2-5p depletion (Figure 6(i)). Overall, these results demonstrate that the circAMOTL1L–miR-92a-2-5p–KLLN axis is involved in RCC growth through regulation of the proliferation and apoptosis of RCC cells.

## 4. Discussion

Accumulating studies have confirmed that the dysregulation of circRNAs contributes to tumor progression [27, 28]. Recently, some circRNAs have been reported to participate in RCC tumorigenesis. However, the function and underlying mechanism of circRNAs in RCC remain largely unknown. In this study, a novel mechanism that circAMOTL1L can go through the circAMOTL1L–miR-92a-2-5p–KLLN axis to suppress RCC growth was discovered.

circAMOTL1L, derived from the circularization of the exon-2 and exon-3 of the Amotl1 gene, was reported to exhibit a tumor suppressor characteristic in prostate cancer by modulating the miR-193a-5p/Pcdha axis to suppress the migration and invasion of PCa cells [25]. The role of circAMOTL1L in RCC development was investigated in this study. We first indicated that circAMOTL1L was notably downregulated in RCC tissues and cell lines, and its level was associated with clinicopathological features and overall survival (Figure 1, Table 1), implying that it may be involved in RCC progression and probably used as a prognostic biomarker for RCC.

The imbalance between cell proliferation and apoptosis is one of the mechanisms underlying tumorigenesis and is associated with the metastasis and drug resistance of tumor cells [29–31]. To explore whether circAMOTL1L exerts regulatory effects on RCC growth, cell function assays were performed both *in vitro* and *in vivo* with a focus on cell proliferation and apoptosis. Our results showed that circAMOTL1L inhibited proliferation and promoted apoptosis in RCC cells. Furthermore, subcutaneous implantation of circAMOTL1L-overexpressing tumor cells decreased the growth ability of the xenograft tumors in nude mice. Moreover, these circAMOTL1L-overexpressing tumor cells exhibit more of an apoptotic phenotype than a proliferative phenotype *in vivo* (Figure 2, Supplementary Figures 1–3). These results suggested that circAMOTL1L acts as an antioncogene in RCC by regulating RCC cell proliferation and apoptosis. In addition, circAMOTL1L may regulate RCC cell migration and invasion. Yang et al. found that decreased circAMOTL1L level facilitates PCa cell migration and invasion, through downregulating E-cadherin and increasing vimentin expression, and leads to EMT and PCa progression [25]. The major limitation of the present study is the lack of evaluation of the effect of circAMOTL1L on the migration and invasion of RCC cells, which needs further investigation. Another limitation is that the rationale underlying the downregulation of circAMOTL1L in RCC is unclear. Yang et al. found that P53 regulated circAMOTL1L expression via RBM25, a RNA binding protein, which directly binds to circAMOTL1L and induces its biogenesis [25]. However, further investigation is also needed to demonstrate the upstream regulatory action of circAMOTL1L in RCC cells.

circRNAs were mostly reported to serve as a miRNA sponge to regulate the function and activity of miRNAs [12, 32]. Particularly, studies have indicated that circRNAs can sponge miRNA to modulate miRNA target gene expression in various cancers. Specifically, Guan et al. found that circPUM1 promoted tumorigenesis and progression of ovarian cancer by sponging miR-615-5p and miR-6753-5p to upregulate NF-*κ*B and MMP2 expression [33]. Chen et al. reported that circPRMT5 promoted tumor metastasis by sponging miR-30c to regulate the expression of snail-1 and E-cadherin in urothelial carcinoma of the bladder [34]. Moreover, circRNA 100146 promoted the proliferation and invasion of cells and inhibited apoptosis by sponging miR-361-3p and miR-615-5p in non-small-cell lung cancer [20]. In this study, oligo pull-down and luciferase reporter assays confirmed that circAMOTL1L could directly bind to miR-92a-2-5p, but circAMOTL1L overexpression could not affect miR-92a-2-5p expression in RCC cells. Notably, whether circRNAs participate in the posttranscriptional regulation of miRNAs remains conflicting. Specifically, circRNA CDR1a harbors 63 conserved miR-7 target sites to serve as the miR-7 sponge but could not affect miR-7 expression [11]. circNCX1 promotes ischemic myocardial injury by sponging miR-133a-3p to inhibit its activity without regulating the miR-133a-3p expression levels [35]. Moreover, accumulating evidence has demonstrated that circRNAs could not affect miRNA degradation but still act as a miRNA sponge and compete for miRNA target sites to suppress its activity. Therefore, our study suggests that circAMOTL1L acts as a “miRNA sponge” of miR-92a-2-5p and is probably uninvolved in miR-92a-2-5p decaying activity (Figure 3).

However, the role of miR-92a-2-5p in RCC has never been studied. We found that miR-92a-2-5p facilitated cell proliferation and suppressed apoptosis in RCC cells and mediated the regulation of circAMOTL1L in the proliferation and apoptosis of RCC cells (Figure 4), indicating that the circAMOTL1L-miR-92a-2-5p pathway plays a regulatory role in RCC development.

circAMOTL1L served as a competing endogenous RNA (ceRNA) for miR-92a-2-5p. miR-92a-2-5p bound by circAMOTL1L could not bind to its target mRNA and lost its ability to inhibit target gene expression. In our study, bioinformatics analysis revealed that KLLN is a potential target of miR-92a-2-5p. Further, luciferase reporter assays and western blotting demonstrated that miR-92a-2-5p could directly bind to the 3′ UTR of KLLN and inhibit its protein expression. At the function level, downregulated KLLN promoted cell proliferation and attenuated apoptosis, which reversed the antiproliferative and proapoptotic effects induced by miR-92a-2-5p knockdown. Alternatively, miR-92a-2-5 improved RCC cell proliferation and reduced apoptosis by targeting KLLN (Figure 5).

KLLN (also called KILLIN), first reported in 2008 which is localized to 10q23 and shares the same transcription start site with PTEN, was indicated as a tumor suppressor necessary and sufficient for p53-mediated apoptosis [26]. Studies have demonstrated that KLLN function is associated with the regulation of cell growth and death. Particularly, KLLN inhibits breast cancer growth by activating p53/p73-mediated apoptosis [36]. In prostate carcinomas, KLLN inhibits tumor cell proliferation and invasiveness by transcriptionally regulating the expression of AR, TP53, and TP73 [3]. Moreover, miR-143-3p promoted proliferation and migration and suppressed apoptosis in vascular smooth muscle cells by targeting KLLN [37]. Observably, methylation significantly downregulated KLLN expression, which led to increased risks of RCC [38]. However, the involvement of KLLN in RCC progression has not yet been explored. Here, we revealed the novel upstream circAMOTL1L–miR-92a-2-5p regulatory pathway for the posttranslational regulation of KLLN. Furthermore, depleted KLLN abolished the antiproliferative and proapoptotic effects induced by circAMOTL1L overexpression in RCC cells. Reinforcing the circAMOTL1L–miR-92a-2-5p–KLLN axis greatly reduced RCC growth *in vivo* (Figure 6, Supplementary Figure 4).

Conclusively, we identified that circAMOTL1L plays an antioncogenic role through the circAMOTL1L–miR-92a-2-5p–KLLN regulatory axis in RCC. circAMOTL1L might sponge miR-92a-2-5p to upregulate KLLN expression, which resulted in suppressing RCC growth by inhibiting proliferation and promoting apoptosis of cells. The novel regulatory network involving the circAMOTL1L–miR-92a-2-5p–KLLN axis might provide a therapeutic strategy for RCC.

## Figures and Tables

**Table 1:** Association of circAMOTL1L expression with clinicopathological characteristics in 51 RCC patients. ^∗^*p* < 0.05 or ^∗∗^*p* < 0.01 was considered significant (chi-square test between 2 groups).

Characteristics	Total	circAMOTL1L expression		*p* value
		Low	High	
Age (years)				0.493
≤60	20	9	11	
>60	31	17	14	
Gender				0.692
Male	34	18	16	
Female	17	8	9	
Tumor stage				0.007
T1+T2	37	11	26	
T3+T4	14	10	4	
Lymph node metastasis				0.006
N0	39	12	27	
N1	12	9	3	
Distant metastasis				0.0462
M0	46	16	30	
M1	5	4	1	

## Data Availability

The raw data used to support the findings of this study are available from the corresponding author upon request.

## Supplementary Materials

Supplementary Materials Supplementary Figure 1: circAMOTL1L depletion facilitated cell proliferation and suppressed apoptosis. Supplementary Figure 2: no phenotypic transformation was induced by circAMOTL1L in HK-2. Supplementary Figure 3: circAMOTL1L overexpression elevated Bax expression and reduced Bcl-2 expression in xenograft tumors, and expression level of Bax was positively correlated with that of circAMOTL1L but expression level of bcl-2 was negatively correlated with that of circAMOTL1L in RCC clinical samples. Supplementary Figure 4: circAMOTL1L overexpression or miR-92a-2-5p depletion increased Bax expression and decreased Bcl-2 expression in the xenograft tumor. Supplementary Table 1: oligos used in this study. Supplementary Table 2: circAMOTL1L harbors 6 supposed binding sites for miR-92a-2-5p.
