# Corrigendum to “circAMOTL1L Suppresses Renal Cell Carcinoma Growth by Modulating the miR-92a-2-5p/KLLN Pathway”

**DOI:** 10.1155/omcl/9898405

**Published:** 2025-09-04

L. Gao, X. Shao, Q. Yue, et al., “circAMOTL1L Suppresses Renal Cell Carcinoma Growth by Modulating the miR-92a-2-5p/KLLN Pathway,” *Oxidative Medicine and Cellular Longevity* 2021 (2021): 9970272, https://doi.org/10.1155/2021/9970272.

In the article, there are errors in Figures 4 and 5. Specifically:

• In Figure 4d, the top right panel (vector + miR-92a-2-5p mimic) of the TUNEL assay is incorrect.
• In Figure 5f, the top-left panel (anti-miR-Ctl + si-Ctl group) and the top-right panel (anti-miR-Ctl + si-KLLN) of the TUNEL assay are both incorrect.

The authors contacted the journal after publication and provided the underlying data for the figures within the article. The authors state that the selection of typical images was made in error when preparing the article for review, and that they identified this when reassessing the raw data following its publication. The statistical results calculated from the raw data and reported within the article are correct and unaffected by this error. The authors apologize for these errors and confirm that they do not affect the conclusions of the article.

Additionally, in Figure 5d, the KLLN band within the western blot was accidentally duplicated in the position of the β-actin band. This error was inadvertently introduced during the production process of the article.

The corrected Figures 4 and 5 are as below:

We apologize for these errors.

## Figures and Tables

**Figure 4:**
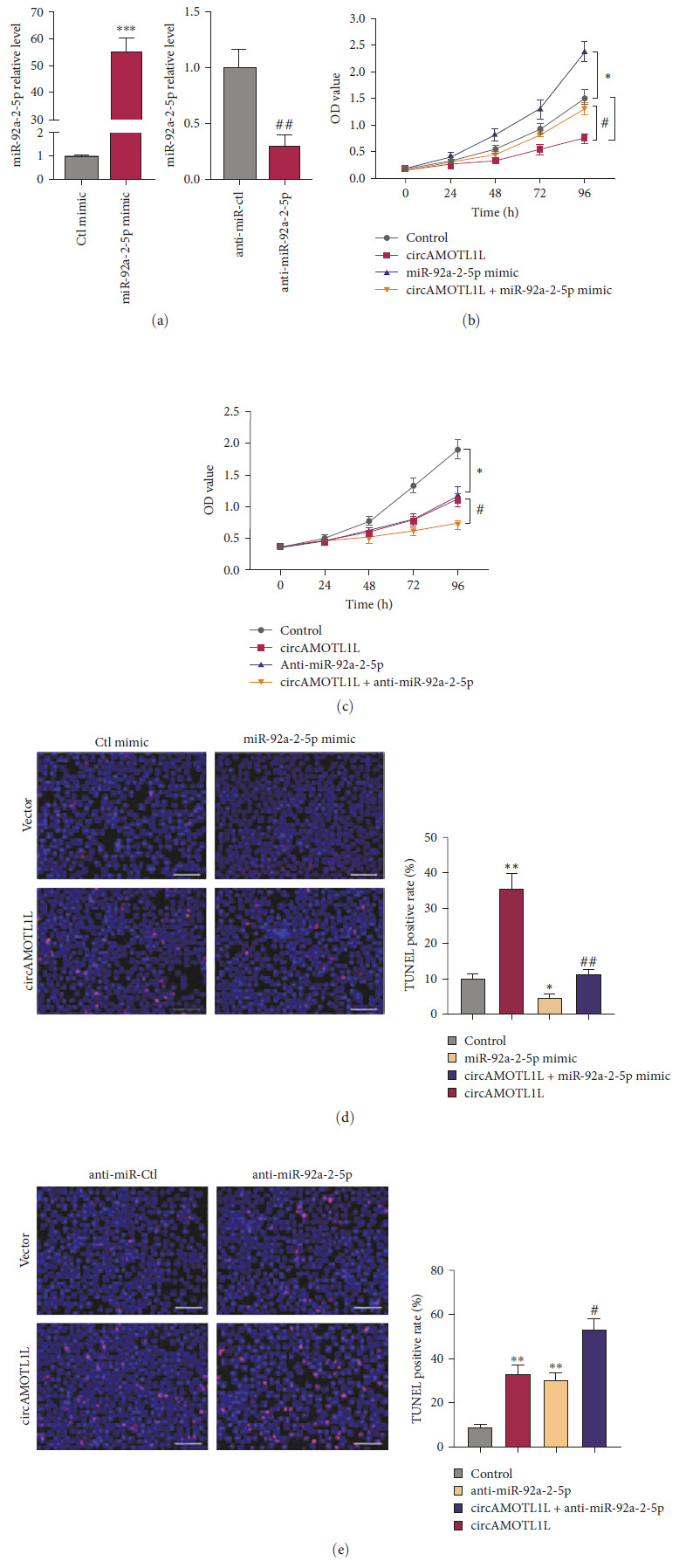
miR-92a-2-5p mediates the regulation of circAMOTL1L in the proliferation and apoptosis of RCC cells. (a) qRT-PCR examined miR-92a-2-5p expression in 786-O cells transfected with the miR-92a-2-5p mimic, anti-miR-92a-2-5p, or its corresponding control miR. *⁣*^*∗∗∗*^*p* < 0 : 001 vs. Ctl mimic; ^##^*p* < 0 : 01 vs. anti-miR-Ctl. (b) CCK-8 assay examined the proliferation of 786-O cells after transfection with pcDNA-circAMOTL1L or miR-92a-2-5p mimic or cotransfection with both. *⁣*^*∗*^*p* < 0 : 05 vs. control group; ^#^*p* < 0 : 05 vs. pcDNA-circAMOTL1L group. (c) CCK-8 assay examined cell proliferation in 786-O cells after transfection with pcDNA-circAMOTL1L or anti-miR-92a-2-5p or cotransfection with both. *⁣*^*∗*^*p* < 0 : 05 vs. control group; ^#^*p* < 0 : 05 vs. pcDNA-circAMOTL1L group. (d, e) TUNEL assay detected cell apoptosis in 786-O cells treated as (b) and (c), respectively. *⁣*^*∗*^*p* < 0 : 05, *⁣*^*∗∗*^*p* < 0 : 01 vs. control group; ^#^*p* < 0 : 05, ^##^*p* < 0 : 01 vs. pcDNA-circAMOTL1L group (scale bars = 50 μm). Graph bars represent mean ± SEM of three independent experiments.

**Figure 5:**
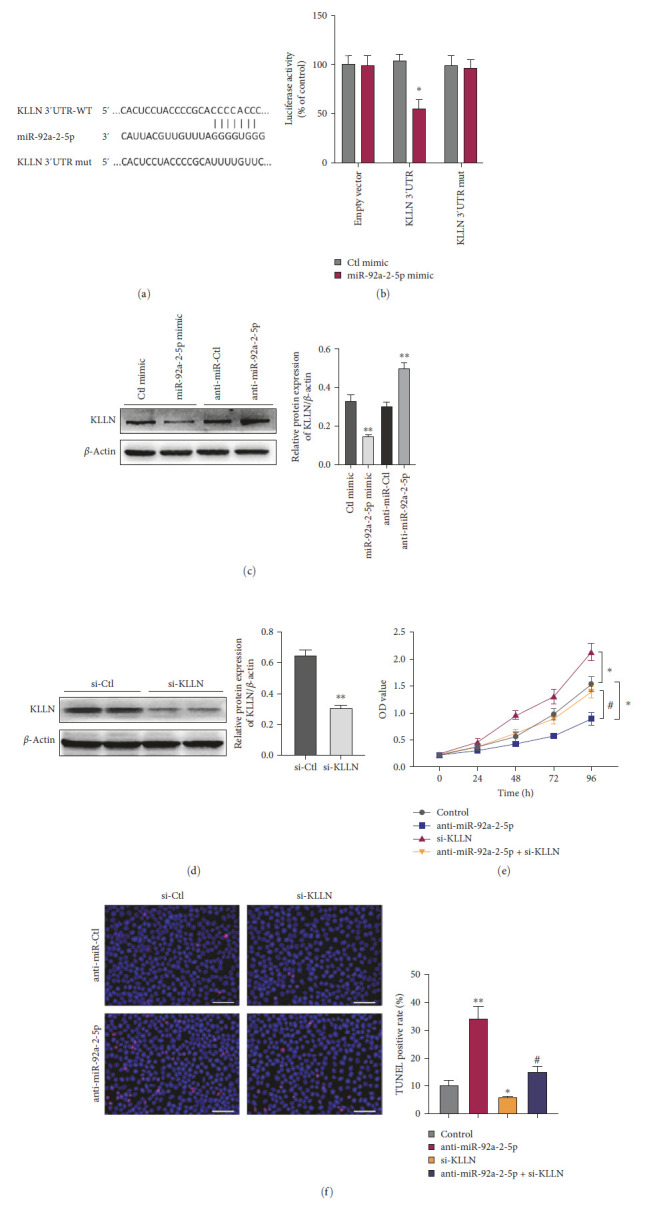
KLLN is a direct target of miR-92a-2-5p in the RCC cell. (a) Prediction of the miR-92a-2-5p binding site in KLLN 3′ UTR. (b) Luciferase reporter assays analyzed luciferase activity in 786-O cells after cotransfecting with the miR-92a-2-5p mimic or Ctl mimic and pmir-GLO vector containing the wild-type or mutated miR-92a-2-5p-binding site (mut) at KLLN 3′ UTR. *⁣*^*∗*^*p* < 0 : 05 vs. Ctl mimic. (c) Western blotting detected KLLN protein levels in 786-O cells transfected with the miR-92a-2-5p mimic, anti-miR-92a-2-5p, or its corresponding control miR. *⁣*^*∗∗*^*p* < 0 : 01 vs. corresponding control groups. (d) Western blotting detected KLLN protein levels in 786-O cells transfected with KLLN-specific siRNA (si-KLLN) or control siRNA (si-Ctl). *⁣*^*∗∗*^*p* < 0 : 01 vs. si-Ctl. (e) CCK-8 assay examined cell proliferation in 786-O cells after transfection with anti-miR-92a-2-5p or si-KLLN or cotransfection with both. *⁣*^*∗*^*p* < 0 : 05 vs. control group; ^#^*p* < 0 : 05 vs. anti-miR-92a-2-5p group. (f) TUNEL assay detected cell apoptosis in 786-O cells treated as (e). *⁣*^*∗*^*p* < 0 : 05, *⁣*^*∗∗*^*p* < 0 : 01 vs. control group; ^#^*p* < 0 : 05 vs. anti-miR-92a-2-5p group (scale bars = 50 μm). Graph bars represent mean ± SEM of three independent experiments.

