# Supplementary material for: circAMOTL1L Suppresses Renal Cell Carcinoma Growth by Modulating the miR-92a-2-5p/KLLN Pathway
Source: Oxid Med Cell Longev. 2021 Oct 4;2021:9970272. doi: 10.1155/2021/9970272 (PMC8505055; doi:10.1155/2021/9970272)
Supplement: Supplementary Materials — Supplementary Figure 1: circAMOTL1L depletion facilitated cell proliferation and suppressed apoptosis. Supplementary Figure 2: no phenotypic transformation was induced by circAMOTL1L in HK-2. Supplementary Figure 3: circAMOTL1L overexpression elevated Bax expression and reduced Bcl-2 expression in xenograft tumors, and expression level of Bax was positively correlated with that of circAMOTL1L but expression level of bcl-2 was negatively correlated with that of circAMOTL1L in RCC clinical samples. Supplementary Figure 4: circAMOTL1L overexpression or miR-92a-2-5p depletion increased Bax expression and decreased Bcl-2 expression in the xenograft tumor. Supplementary Table 1: oligos used in this study. Supplementary Table 2: circAMOTL1L harbors 6 supposed binding sites for miR-92a-2-5p. [file 9970272.f1.docx]

Supplementary figures

Supplementary Fig.1. circAMOTL1L depletion facilitated cell proliferation and suppressed apoptosis.

a:

b:


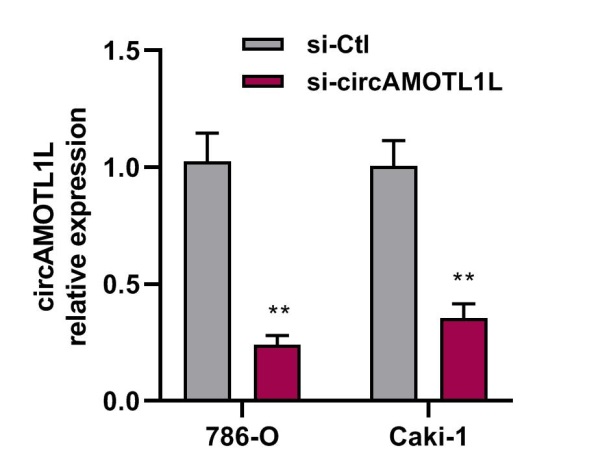

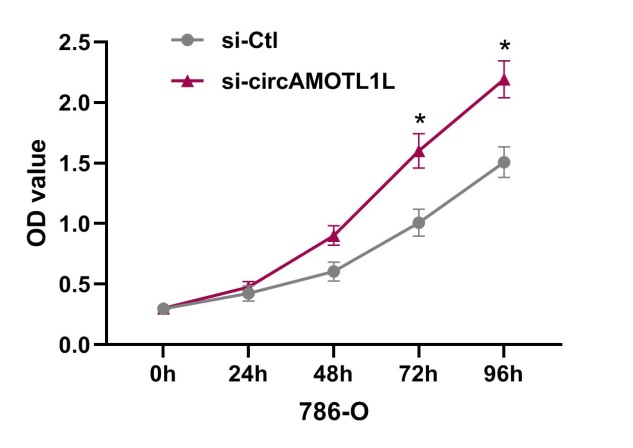

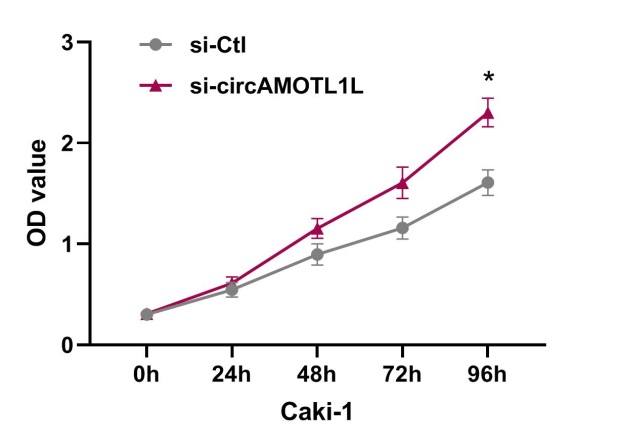


c:


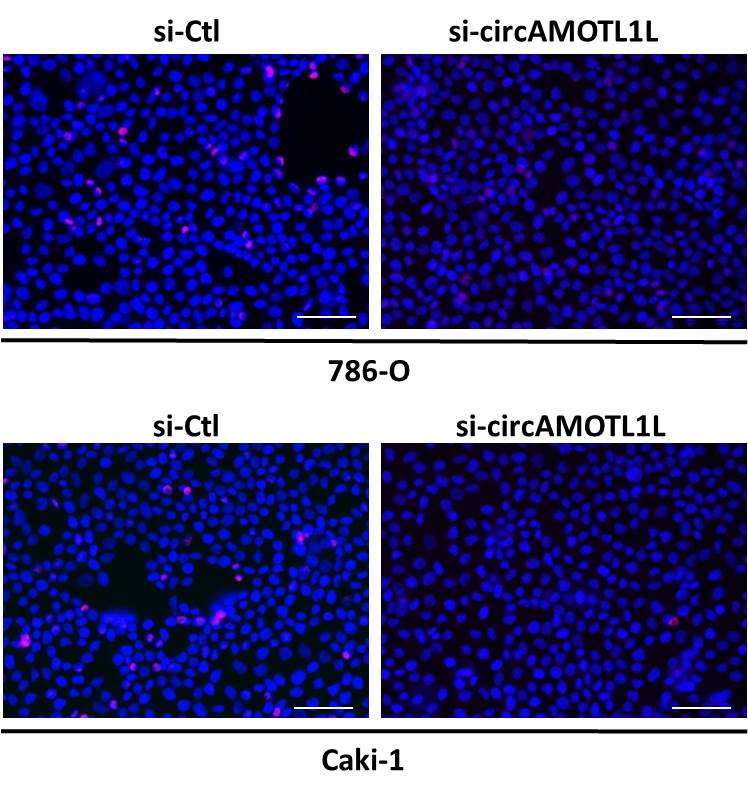


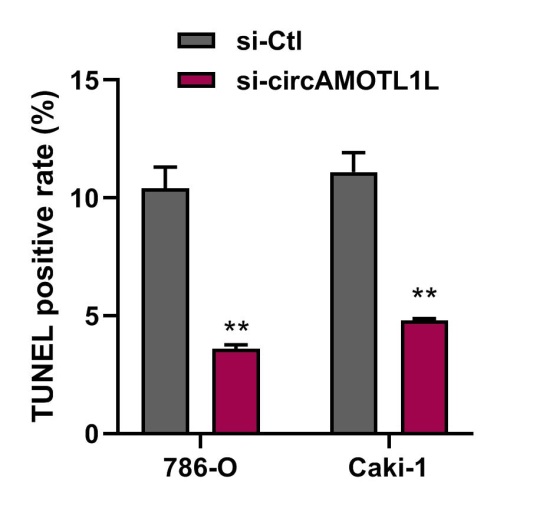


**Supplementary Fig.1.** circAMOTL1L depletion facilitated cell proliferation and suppressed apoptosis. a. qRT–PCR examined the circAMOTL1L expression level in 786-O and Caki-1 cells after transfected with si-circAMOTL1L or si-Ctl. ** *p* < 0.01 vs. si-Ctl. b. CCK-8 assay examined the cell proliferation in 786-O and Caki-1 cells transfected with si-circAMOTL1L or si-Ctl. * *p* < 0.05 vs. si-Ctl. **c.** TUNEL assay detected cell apoptosis in 786-O and Caki-1 cells after transfecting with si-circAMOTL1L or si-Ctl. ** *p* < 0.01 vs. si-Ctl (Scale bars = 50 μm).

Supplementary Fig.2. No phenotypic transformation was induced by circAMOTL1L in HK-2.


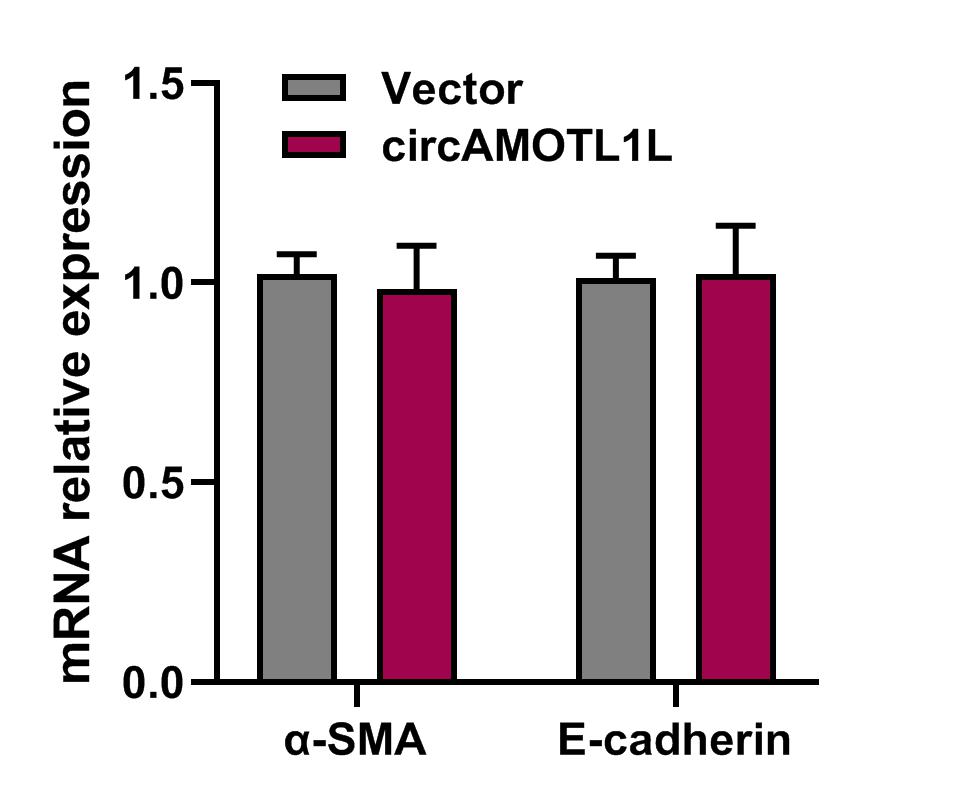


a:


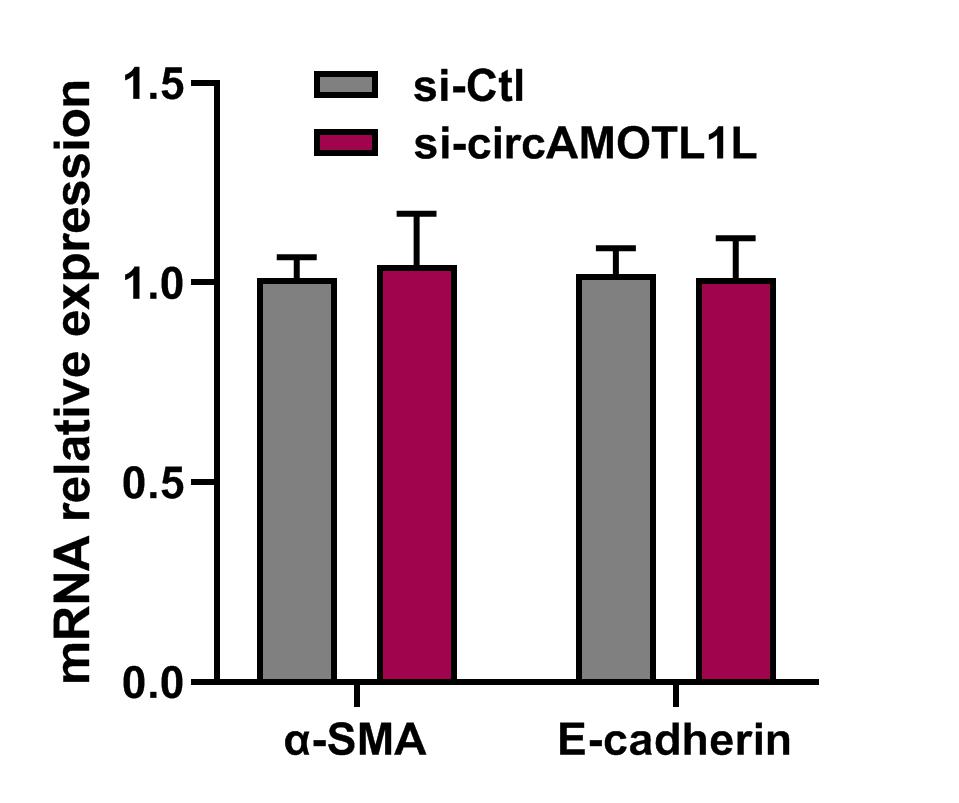


b:

**Supplementary Fig. 2.** No phenotypic transformation was induced by circAMOTL1L in HK-2. **a.** qRT–PCR examined α-SMA and E-cadherin in HK-2 expression after transfecting with pcDNA-circAMOTL1L or vector control. **b.** qRT–PCR examined α-SMA and E-cadherin in HK-2 expression after transfecting with si-circAMOTL1L or si-Ctl.

Supplementary Fig.3. circAMOTL1L overexpression elevated Bax expression and reduced Bcl-2 expression in xenograft tumors, and expression level of Bax was positively correlated with that of circAMOTL1L but expression level of bcl-2 was negatively correlated with that of circAMOTL1L in RCC clinical samples.

a:


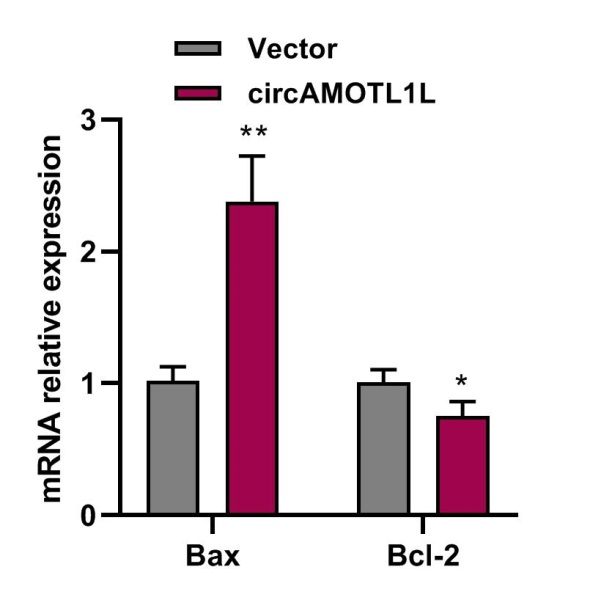


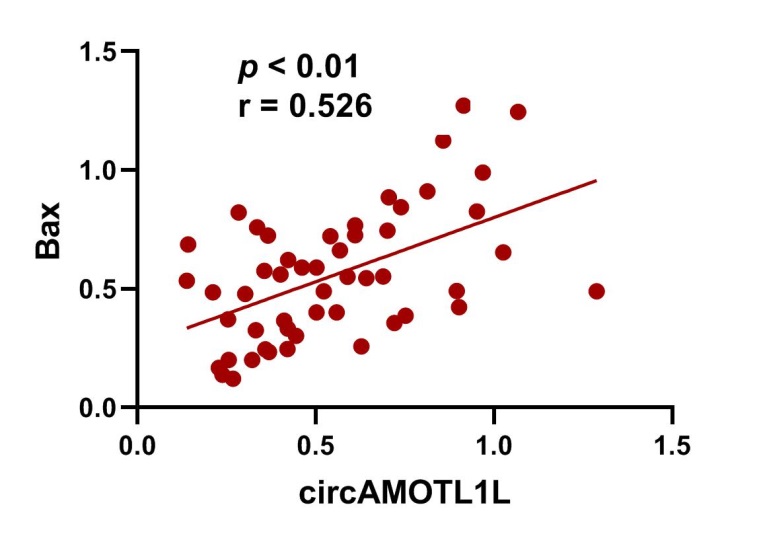

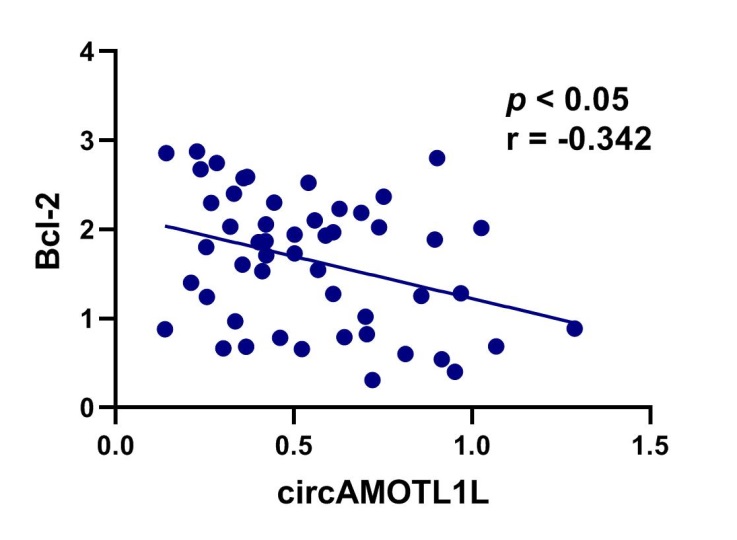


c:

b:

**Supplementary Fig.3.** circAMOTL1L overexpression elevated Bax expression and reduced Bcl-2 expression in xenograft tumors, and expression level of Bax was positively correlated with that of circAMOTL1L but expression level of bcl-2 was negatively correlated with that of circAMOTL1L in RCC clinical samples. **a.** qRT–PCR examined Bax and Bcl-2 expression in xenograft tumors. **p* < 0.05, ***p* < 0.01 vs. LV-Ctl (n = 6 in each group). **b-c.** Pearson correlation analyzed the relationship between Bax mRNA and circAMOTL1L (*p* < 0.01, r=0.526), and Bcl-2 mRNA and circAMOTL1L (*p* < 0.05, r=-0.342).

Supplementary Fig.4. circAMOTL1L overexpression or miR-92a-2-5p depletion increased Bax expression and decreased Bcl-2 expression in xenograft tumor.

a:

b:


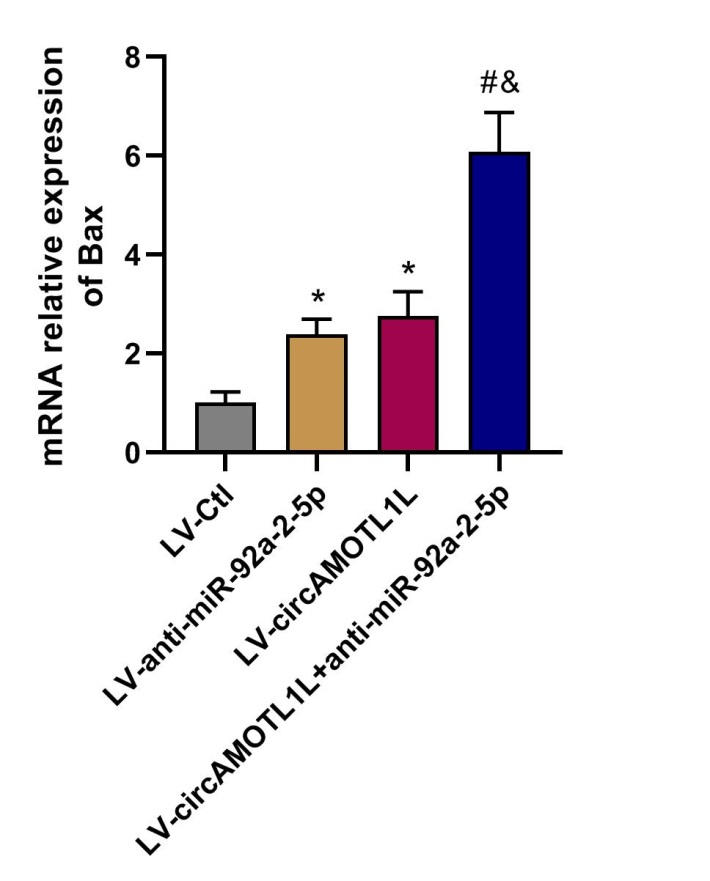

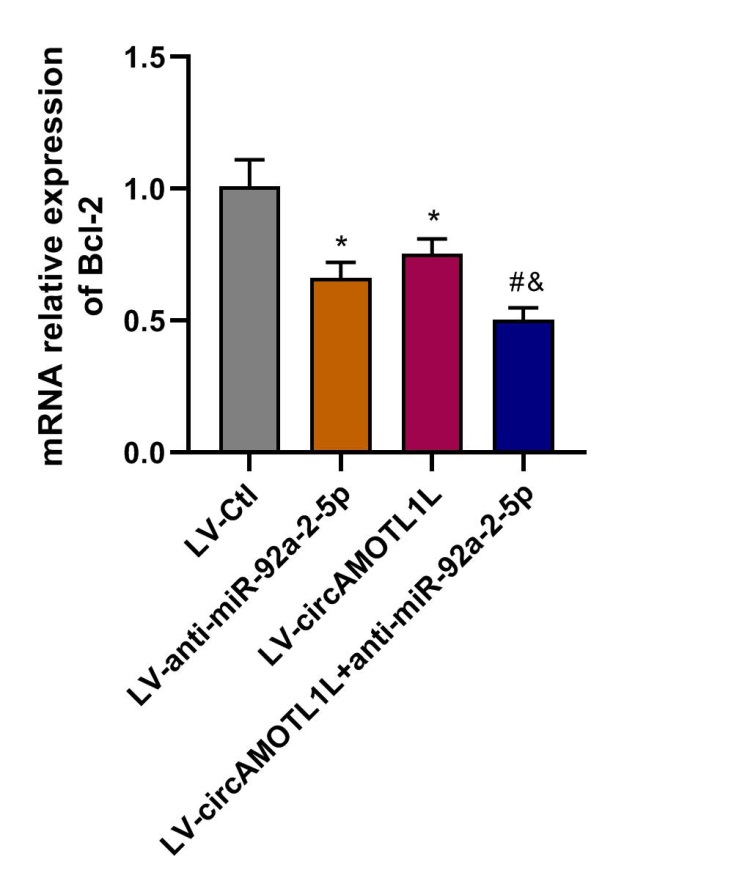


**Supplementary Fig.4.** circAMOTL1L overexpression or miR-92a-2-5p depletion increased Bax expression and decreased Bcl-2 expression in xenograft tumor. **a-b.** qRT–PCR examined Bax and Bcl-2 expression in xenograft tumors. **p* < 0.05 vs. LV-Ctl; ^#^*p* < 0.05 vs. LV-circAMOTL1L; ^&^*p* < 0.05 vs. LV-anti- miR-92a-2-5p.

Supplementary Table 1. Oligos used in this study

| **Oligo name** | **Sequence (5' - 3')** | **Purpose** |
| --- | --- | --- |
| circAMOTL1L-F | CCACGAGATGGTCAAGCCCTAC | qRT-PCR for circAMOTL1L. |
| circAMOTL1L-R | CCACCGTCAAAGCAGATGTTTC |  |
| GAPDH-F | AAGGTGAAGGTCGGAGTCAAC | qRT-PCR for GAPDH |
| GAPDH-R | GGGGTCATTGATGGCAACAATA |  |
| pcDNA-AMOTL1-inf-F | CATTCCCTTTCTTTCCCTCAGGGTCCCCTTCTGCTTGTTATAGCCC | Clone the overexpression vector for circAMOTL1L |
| pcDNA-AMOTL1-inf-R | CGTCTGCAGTTGATACTCACCTCGTTACCCCATACTCAGGGGGTG |  |
| Biotin-con | CTTCACCCCAGGAACAAACTCCTTTGCATT | Oligo-pulldown NC Biotin |
| circAMOTL1L-biotin | CTATAACAAGCAGAAGGGGACCCTCGTTAC | Oligo-pulldown |
| GLO-circAMOTL1L-1072-F | GCTCGCTAGCCTCGAGGGTCCCCTTCTGCTTGTTATAGCC | Pmir-GLO-circAMOTL1L vector for Dual-Glo Luciferase Assay |
| GLO-circAMOTL1L-1072-R | ATGCCTGCAGGTCGACCTCGTTACCCCATACTCAGGGG |  |
| miR-92a-2-5p-F | CGGGGGTGGGGATTTGTTGC | qRT-PCR for miR-92a-2-5p |
| miR-23a-5p-F | CGTGGGTTCCTGGCATGCTG | qRT-PCR for miR-23a-5p |
| miR-125a-5p-F | CGTCCCTGAGACCCTTTAACCTGTG | qRT-PCR for miR-125a-5p |
| miR-149-3p-F | AGGGAGGGACGGGGGCTG | qRT-PCR for miR-149-3p |
| miR-193a-5p-F | CTGGGTCTTTGCGGGCGAG | qRT-PCR for miR-193a-5p |
| miR-193b-5p-F | GTCGGGGTTTTGAGGGCGAG | qRT-PCR for miR-193b-5p |
| miR-339-5p-F | GCCTCCCTGTCCTCCAGGAGC | qRT-PCR for miR-339-5p |
| miR-345-3p-F | GCCCTGAACGAGGGGTCTGG | qRT-PCR for miR-345-3p |
| miR-370-3p-F | GCCTGCTGGGGTGGAACCTG | qRT-PCR for miR-370-3p |
| miR-378a-5p-F | GGCTCCTGACTCCAGGTCCTGTG | qRT-PCR for miR-378a-5p |
| miR-485-5p-F | AGAGGCTGGCCGTGAT | qRT-PCR for miR-485-5p |
| miR-513a-5p-F | GGCCTTCACAGGGAGGTGTCAT | qRT-PCR for miR-513a-5p |
| miR-612-F | GCTGGGCAGGGCTTCTGAGC | qRT-PCR for miR-612 |
| miR-762-F | GGGGCTGGGGCCGGGGCC | qRT-PCR for miR-762 |
| miR-943-F | CTGACTGTTGCCGTCCTCCAG | qRT-PCR or miR-943 |
| miR-1229-5p-F | GTGGGTAGGGTTTGGGGGAGAG | qRT-PCR for miR-1229-5p |
| RNU6-1(U6)-F | GTGCTCGCTTCGGCAGCACATATAC | qRT-PCR for U6 |
| RNU6-1(U6)-R | AAAATATGGAACGCTTCACGAATTTGC |  |
| miR-92a-2-5p-biotin | GGGTGGGGATTTGTTGCATTAC | Oligo-pulldown |
| miR-193a-5p-biotin | TGGGTCTTTGCGGGCGAGATGA | Oligo-pulldown |
| miR-339-5p-biotin | TCCCTGTCCTCCAGGAGCTCACG | Oligo-pulldown |
| P53-F | CCAGATGAAGCTCCCAGAATGCC | qRT-PCR for P53 |
| P53-R | CTGTCCCAGAATGCAAGAAGCCC |  |
| PCNA-F | CAAGTGGAGAACTTGGAAATGG | qRT-PCR for PCNA |
| PCNA-R | CTATGGTAACAGCTTCCTCCT |  |
| Bax-F | GCTTCAGGGTTTCATCCAG | qRT-PCR for Bax |
| Bax-R | GGCGGCAATCATCCTCTG |  |
| Bcl-2-F | TACTTAAAAAATACAACATCACAG | qRT-PCR for Bcl-2 |
| Bcl-2-R | GGAACACTTGATTCTGGTG |  |
| si-circAMOTL1L-F | CGAGGGUCCCCUUCUGCUUTT | Knock-down circAMOTL1L |
| si-circAMOTL1L-R | AAGCAGAAGGGGACCCUCGTT |  |

Supplementary Table 2. circAMOTL1L harbors 6-supposed binding sites for miR-92a-2-5p

| **microRNA** | **position** | **matching** |
| --- | --- | --- |
| miR-92a-2-5p | 39 | CAGGTTCTAGAAGACTCCACCT  \| \|\|: \|:\|\|\|\|\|: CATTACGTTGTTTAGGGGTGGG |
| miR-92a-2-5p | 331 | GGAGTGCAGGACCAGCCCATCC  \|\|\|: \| \|\|\|\|:\|\| CATTACGTTGTTTAGGGGTGGG |
| miR-92a-2-5p | 364 | TTTCTTCCACGGAAAACCTCACTC  \|\|::\| \|\|:\|\|\|:\| CATTACGTTGTTT--AGGGGTGGG |
| miR-92a-2-5p | 500 | GAACAACGAGGAACTGCCCACTT  \|:\| :\|\| \| \|\|\|\|\|:: CATTACGTTGTTT-AGGGGTGGG |
| miR-92a-2-5p | 774 | GCCAAGCAACACCTTCCCGGCTC  \|\|\|\|\|\| \|\|\|\| :\|:\| CATTACGTTGT-TTAGGGGTGGG |
| miR-92a-2-5p | 39 | ACCAAGCAAATGATGTCCCCAGTC  \|\|\|\| :\|\|\|\|\|\| :\| CATTACGTT--GTTTAGGGGTGGG |
